# Engineering and validation of a dual luciferase reporter system for quantitative and systematic assessment of regulatory sequences in Chinese hamster ovary cells

**DOI:** 10.1038/s41598-022-09887-2

**Published:** 2022-04-11

**Authors:** Yagmur Toktay, Bengisu Dayanc, Serif Senturk

**Affiliations:** 1 Izmir Biomedicine and Genome Center, Izmir, 35340 Turkey; 2 Department of Genome Sciences and Molecular Biotechnology, Izmir International Biomedicine and Genome Institute, Dokuz Eylul University, Izmir, 35340 Turkey

**Keywords:** Expression systems, Molecular engineering, Biotechnology, Molecular biology

## Abstract

Ongoing research efforts to identify potent regulatory sequences that deliver robust and sustained transgene expression are critical for Chinese hamster ovary (CHO) cell line development technologies to meet the growing demand for recombinant proteins. Here we report the engineering and validation of a highly customizable single vector toolkit that comprises an all-in-one dual luciferase reporter system for quantitative and systematic interrogation of transcriptional regulatory sequences in transient and stable transfectants of CHO cells. To model the execution of the reporter system, we implemented a battery of known constitutive promoters including human CMV-mIE, SV40, HSV-TK, mouse PGK, human EF1α, EF1α short (EFS), human UBC, synthetic CAG, and Chinese hamster EF1α (CHEF1α). Of the nine promoters, CMV-mIE yielded the highest transcriptional activity in transient transfection settings, while CHEF1α was the strongest among a select subset of promoters in stable transfectants of CHO-DG44 pools. Remodeling the vector toolkit to build a dual fluorescent reporter system featured an alternative to bioluminescence based reporters. We infer that the findings of this study may serve as a basis to establish new vectors with weak or strong constitutive promoters. Furthermore, the modular all-in-one architecture of the reporter system proved to be a viable tool for discovering novel regulatory sequences that ensure high levels of transient and stable transgene expression in CHO and perhaps other mammalian cell lines.

## Introduction

The advent of recombinant DNA technology enabled the propagation of genetic material from diverse organisms and their incorporation into a host cell genome. This technology has been widely used in molecular and pharmaceutical biotechnology to engineer host cell lines and produce clinically relevant recombinant proteins such as hormones, enzymes, and monoclonal antibodies^1^. At present, mammalian expression systems constitute the most prevalent source of complex multimeric proteins^2^. Among these systems, Chinese hamster ovary (CHO) cells remain the primary expression platform^2,3^. Furthermore, the most intensive research field in recombinant CHO cell line development focuses on establishing clonal cells with robust and stable transgene expression^4^. In this multistep process, a DNA expression vector encoding the foreign protein is initially delivered into CHO cells, followed by a selection process to isolate stable transfectants with integration of expression cassettes into their genome^5,6^. An optimized architecture and robust genetic elements are imperative to a successful CHO expression vector^7,8^. Transcriptional regulatory sequences such as promoters, enhancers, and other cis-acting elements are among the key vector components that significantly impact the level of forced transgene expression, hence the success of an expression vector^7,9^. Based on this premise, the careful selection of transcriptional regulatory sequences that induce robust and stable transgene expression in CHO cells may have a profound effect on the development of effective transgene expression cassettes and vectors.

Genetic reporter systems are powerful molecular tools that can assess the strength of regulatory DNA sequences^10^. Conventional dual vector reporter systems employ two monocistronic vectors and require cotransfection to express the experimental and the internal reporter separately, which serves as a normalization control^11,12^. Normalization is a critical step for mitigating the effects of variables such as cell number, viability, transfection efficiency and DNA copy number, all of which may impose confounding effects on the level of reporter gene expression. Despite their widespread utility in mammalian cell cultures, including CHO cells, these systems may inherently suffer from certain limitations such as skewing during normalization, resulting in poor accuracy or experimental bias^12^. Furthermore, they may be time-consuming and labor-intensive to implement, making them inefficient tools for quantitative and systematic analysis of known or potentially novel regulatory sequences with robust and stable expression activity in CHO cells. Based on this premise, we present here the engineering, validation, and applicability of a novel and highly customizable all-in-one genetic vector toolkit. The single vector design entails a dual luciferase (also a dual promoter) reporter system with an invariable regulatory sequence (a viral promoter) controlling the expression of an internal reporter, Renilla luciferase (Rluc), and a variable regulatory sequence (a collection of known promoters) modulating the expression of Firefly luciferase (Fluc). In addition to regulatory sequences, the bioluminescence reporters can be tailored to specific requirements, justifying the versatility of the vector backbone. Specifically, we were able to remodel the dual luciferase reporter system into a dual fluorescent reporter system by successfully replacing Fluc and Rluc coding sequences with enhanced green fluorescence (eGFP) and tandem dimer Tomato (tdTomato) fluorescent reporter genes, respectively. To establish the utility of the reporter system, we modeled an experimental scheme based on systematic and quantitative comparison of a compendium of existing constitutive promoters in two contexts: (1) transient transfection of different CHO cell variants as well as HEK-293T cells, and (2) stable cultures of CHO-DG44 suspension cells. The promoters tested herein include human cytomegalovirus (CMV) major immediate early (mIE) enhancer-containing promoter (CMV-mIE), simian virus 40 enhancer/early promoter (SV40), herpes simplex virus (HSV) thymidine kinase (TK) promoter (HSV-TK), mouse phosphoglycerate kinase 1 promoter (PGK), human eukaryotic translation elongation factor 1 alpha promoter (EF1α), EF1α short promoter (EFS), human ubiquitin C promoter (UBC), CMV early enhancer/chicken beta-actin promoter (CAG), and Chinese hamster eukaryotic translation elongation factor 1 alpha promoter (CHEF1α). We expect that the results presented here could potentially serve as a point of reference to guide life science researchers in creating new vectors with their choice of constitutive promoters. More importantly, the highly customizable architecture of the vector toolkit can set a benchmark for the discovery of novel regulatory sequences that achieve high transient and stable transgene expression in CHO and possibly other mammalian cell cultures.

## Results

### Experimental workflow and vector designs

**Figure 1:**
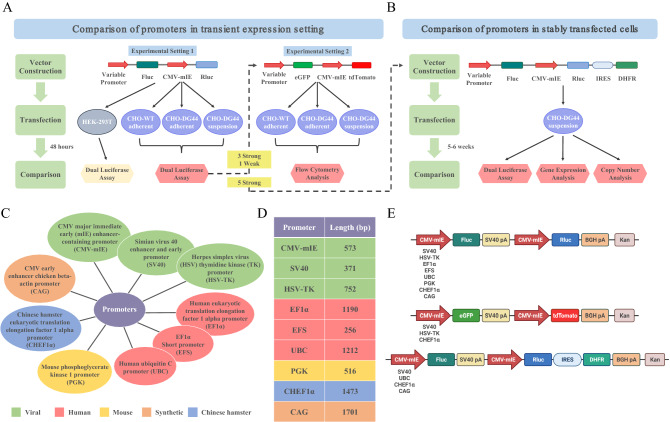
Experimental workflow of the study, promoter characteristics and vector designs. (**A**,**B**) Schematic representation of the workflow: Promoter strengths in the transient expression context were quantified using dual luciferase assay and flow cytometry analysis. (**A**) All nine promoters were compared based on luciferase activity in CHO cell variants and HEK-293T cells (Experimental setting 1). To corroborate the dual luciferase reporter findings, one weak and three relatively strong promoters were cloned into the dual fluorescence reporter system and further investigated by flow cytometry analysis (Experimental setting 2). (**B**) The activity of five relatively strong promoters selected and prioritized from (**A**) was evaluated in stable transfectants of CHO-DG44 suspension cells by dual luciferase assay. Further experiments were performed to analyze the expression of Fluc and Rluc genes, as well as the copy number of Rluc and DHFR genes. (**C**) The list of well-known constitutive promoters tested herein. Color codes represent the origin of promoters. (**D**) The table depicts promoter lengths. (**E**) Representative vector maps of all-in-one reporter systems. In the dual luciferase reporter system, the variable promoter (CMV-mIE) was replaced by SV40, HSV-TK, EF1α, EFS, UBC, PGK, CHEF1α and CAG promoters. In the dual fluorescent reporter system, the variable CMV-mIE was replaced by SV40, HSV-TK and CHEF1α promoters. For stable expression analysis, the constructs with CMV-mIE, SV40, UBC, CHEF1α and CAG promoters were engineered to coexpress Rluc and DHFR genes separated by an IRES sequence, creating a bicistronic expression cassette. The schematics in (**E**) are created with BioRender.com.

One way to validate the all-in-one reporter system is through transient or stable transfection of mammalian cells. Within this framework, the schematics in Fig. 1 illustrate the overall workflow of this study and feature the characteristics of promoters. To summarize, the study involves the engineering and validation of a reporter system containing nine common promoters with functionally and structurally diverse attributes: three viral promoters (CMV-mIE, SV40, and HSV-TK), three human promoters (EF1α, EFS, and UBC), one synthetic promoter (CAG), one mouse promoter (PGK), and one Chinese hamster promoter (CHEF1α) (Fig. 1C,D). The vector collection also features an empty control reporter lacking any variable promoter sequence (NP: no promoter). To experimentally verify the dual luciferase reporter system, first we comparatively investigated the transcriptional regulatory activities of the promoter panel in a transient transfection setting of CHO cell variants and HEK-293T cells (Fig. 1A, Experimental setting 1). Next, we reconstructed the reporter system to coexpress eGFP and tdTomato fluorescent reporters, with a focus on a subset of promoters that were selected and prioritized from the prior analysis (Fig. 1A, Experimental setting 2). Finally, we demonstrated that with further engineering the dual luciferase reporter system could be employed to quantify the strength of regulatory sequences in stable transfectants of CHO-DG44 suspension cells (Fig. 1B). Of particular relevance, CHO-DG44 cell line exhibits homozygous deletion of the entire dihydrofolate reductase (DHFR) locus, a feature routinely exploited for auxotrophic selection and transgene copy number amplification^13–16^. Finally, a detailed schematic of the modular vector backbones used throughout this study is rendered in Fig. 1E.

### Transient transfection of the dual luciferase reporter system

**Figure 2:**
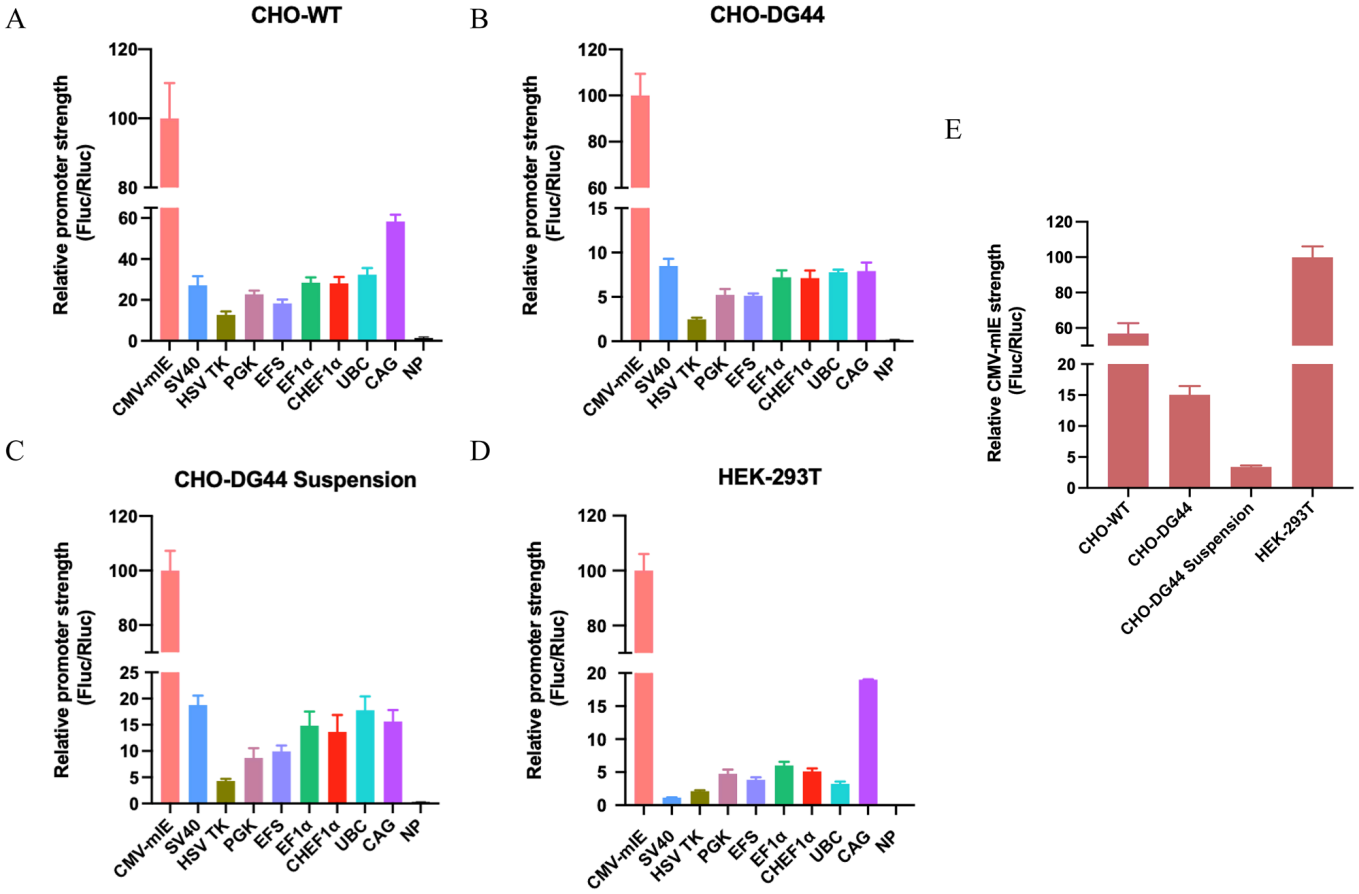
Analysis of promoter strength using the dual luciferase reporter system in transient expression settings. The activities of nine promoters was evaluated by dual luciferase assay at 48 h post-transfection. Fluc expression is driven by the variable promoter and enables the systematic comparison of promoter activities, while the Rluc signal is an internal control regulated by the invariable CMV-mIE promoter. The measurement of promoter strength based on the ratio of Fluc to Rluc signals was investigated in (**A**) CHO-WT, (**B**) CHO-DG44 adherent, (**C**) CHO-DG44 suspension, and (**D**) HEK-293T cell lines. The CMV-mIE promoter containing vector exhibited the highest value which was normalized to 100 in all cell lines. (**E**) Cross comparison of promoter activities across cell lines was examined by setting the CMV-mIE promoter as a standard. The relative activity in HEK-293T cell line was set at 100.

Transient transfection technique in which the foreign DNA does not integrate into the host genome enables simple, quick, and cost-effective analysis of transgene expression^17^. This experimental scheme would allow for rapid validation of the bioluminescence reporter toolkit and provide an early assessment of promoter activities (Fig. 1A). To this end, we individually transfected CHO-WT, CHO-DG44 adherent, and CHO-DG44 suspension cells with the vector panel using an optimized protocol with a 1:3 mass-to-volume ratio of plasmid DNA to Lipofectamine 3000. We achieved comparable transfection efficiencies in CHO-WT and CHO-DG44 adherent cell lines with pEGFP-N1, a well-known plasmid that expresses the eGFP fluorescent protein. However, CHO-DG44 suspension cells had a lower efficiency with this protocol (data not shown). At 48 h post transfection, we performed the dual luciferase reporter assay to quantify the activities of each promoter. We estimated and correlated promoter strengths by calculating the ratio of Fluc to Rluc bioluminescence signals. Of the nine promoters, the CMV-mIE promoter displayed the highest activity (normalized to 100) in CHO-WT cells, followed by CAG (58.3), UBC (32.3), EF1α (28.5), CHEF1α (28.1), and SV40 (27.1) promoters, while the activities of PGK (22.7), EFS (18.28), and HSV-TK (12.7) promoters were relatively weaker (Fig. 2A). Intriguingly, the overall activity profile of other promoters was lower in CHO-DG44 adherent and CHO-DG44 suspension cells as opposed to CMV-mIE. In particular, the promoter activities in CHO-DG44 adherent cells were as the following: SV40 (8.5), CAG (7.9), UBC (7.8), EF1α (7.2), CHEF1α (7.1), PGK (5.2), EFS (5.1), and HSV-TK (2.4) (Fig. 2B). Similarly, the strongest promoter in CHO-DG44 suspension cells was CMV-mIE, which was followed by SV40 (18.8), UBC (17.8), CAG (15.6), EF1α (14.8), CHEF1α (13.7), EFS (9.9), PGK (8.7), and HSV-TK (4.3) promoters (Fig. 2C). The Fluc signal of the NP construct was nearly undetectable, proving that the Fluc expression was selectively driven by the variable promoters. These results indicate that under transient transfection conditions the CMV-mIE promoter is the strongest in CHO cell variants. In contrast, the remaining promoters deliver less and slightly variable activities in CHO-DG44 adherent and CHO-DG44 suspension cells when compared to CHO-WT data, an important finding that warrants further investigation.

Next, we sought to establish additional evidence demonstrating that the dual luciferase reporter system can have a widespread utility in mammalian cell cultures. To that purpose, we investigated the relative strength of promoters in HEK-293T, a highly transfectable variant of HEK-293 cell line that expresses a temperature-sensitive mutant of the SV40 T antigen. In essence, the human embryonic kidney 293 (HEK-293) cell line and its variants are particularly attractive mammalian host systems for recombinant protein production^18,19^. In close agreement with the CHO data, we found that the CMV-mIE promoter had the greatest activity (Fluc/Rluc) in HEK-293T cells. This was followed by CAG (19.0), EF1α (6.0), CHEF1α (5.1), PGK (4.7), EFS (3.8), UBC (3.2), and HSV-TK (2.1), SV40 (1.1) promoters. As expected, the NP construct had no detectable Fluc activity (Fig. 2D). Lastly, we set out to provide a comparative analysis of Fluc/Rluc ratios across cell lines, using the CMV-mIE promoter activity as a reference. In this case, HEK-293T cells exhibited the strongest activity (normalized to 100), while the lowest activity was observed in CHO-DG44 suspension cells (Fig. 2E). Using this information, the remaining promoters may be cross compared across four different cell lines. Taken together, these results successfully validate the all-in-one dual luciferase reporter system and offer compelling insights that this single vector toolkit can be utilized in transient expression settings for rapid and systematic assessment of promoter strength and perhaps other regulatory sequences in CHO and HEK-293 cell lines, and their variants. This would most likely be extended to other mammalian host cell lines.

### Dual fluorescence reporter system in transient expression setting

**Figure 3:**
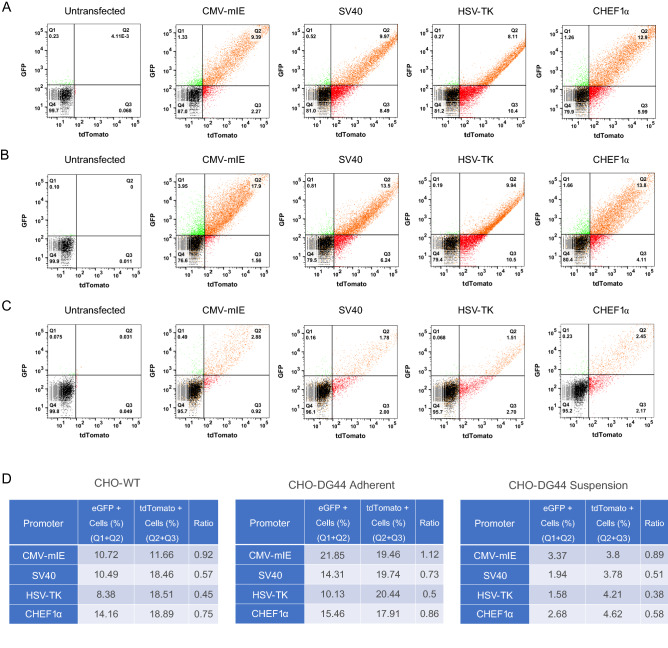
Cross validation of bioluminescence based results and analysis of promoter strength using the dual fluorescence reporter system in transient expression settings. Promoter strength was investigated by flow cytometry to verify bioluminescence based analyses while also demonstrating that the reporter system can function with fluorescence signals. The vector panel carrying CMV-mIE, SV40, HSV-TK and CHEF1α promoters was transiently transfected into CHO cell variants. At 48 h post-transfection, flow cytometry was conducted to evaluate promoter activities on (**A**) CHO-WT, (**B**) CHO-DG44 adherent and (**C**) CHO-DG44 suspension cells. (**D**) Similar to the dual luciferase reporter system, the ratio of eGFP/tdTomato fluorescence signals provides a systematic and quantitative comparison of the promoters. The strength of each promoter was expressed as the ratio of eGFP positive cells (Q1 + Q2) divided by the percentage of tdTomato positive cells (Q2 + Q3).

After verifying the utility of the dual luciferase reporter system in CHO cell variants and HEK-293T cells, we attempted to expand the engineering capacity of the modular vector toolkit. To that goal, we specifically remodeled the dual luciferase vector backbone into an all-in-one dual fluorescence reporter system through extensive molecular cloning, substituting the Fluc and Rluc genes with eGFP and tdTomato coding sequences, respectively. Just like the dual luciferase reporter constructs, the new all-in-one system also provides simultaneous expression of the fluorescent reporter genes. Specifically, while tdTomato expression is controlled by the invariable CMV-mIE promoter, eGFP expression is dictated by the variable promoters, specifically CMV-mIE, SV40, CHEF1α, and HSV-TK (Fig. 1E). To examine promoter activities, we transiently transfected the vector panel into CHO-WT, CHO-DG44 adherent and CHO-DG44 suspension cells. At 48 h post-transfection, we performed flow cytometry analysis to quantitatively monitor eGFP and tdTomato reporter signals. Again, much like the previous calculations, the ratio of total eGFP-positive cells to total tdTomato-positive cells (eGFP/tdTomato) was expressed as a surrogate parameter of promoter strength. In consequence, we found that CHO-WT cells transfected with the CMV-mIE promoter-containing vector had the greatest ratio for eGFP/tdTomato (0.92), followed by CHEF1α (0.75) and SV40 (0.57) promoters, while the HSV-TK promoter exhibited the lowest value (0.45) for eGFP/tdTomato ratio (Fig. 3A,D). When compared to CHO-WT cells, CHO-DG44 adherent cells displayed the same order of promoter activities however with slight differences in eGFP/tdTomato ratios. Specifically, the CMV-mIE promoter had a ratio of 1.12, followed by CHEF1α (0.86), SV40 (0.73), and HSV-TK (0.50) promoters (Fig. 3B,D). Despite having a significantly lower transfection efficiency, CHO-DG44 suspension cells also exhibited the same order of promoter strength, with the eGFP/tdTomato ratios being 0.89 for CMV-mIE, 0.58 for CHEF1α, 0.51 for SV40, and 0.38 for HSV-TK promoters (Fig. 3C,D). In summary, we conclude that the dual fluorescent reporter system may offer an alternative to the bioluminescence based reporters for monitoring the strength of regulatory sequences. Besides, these data fortunately corroborate the versatility of the vector backbone, implying that further modifications could be tailored to suit individual requirements or specifications.

### Dual luciferase reporter system in CHO-DG44 stable polyclonal cell pools

**Figure 4:**
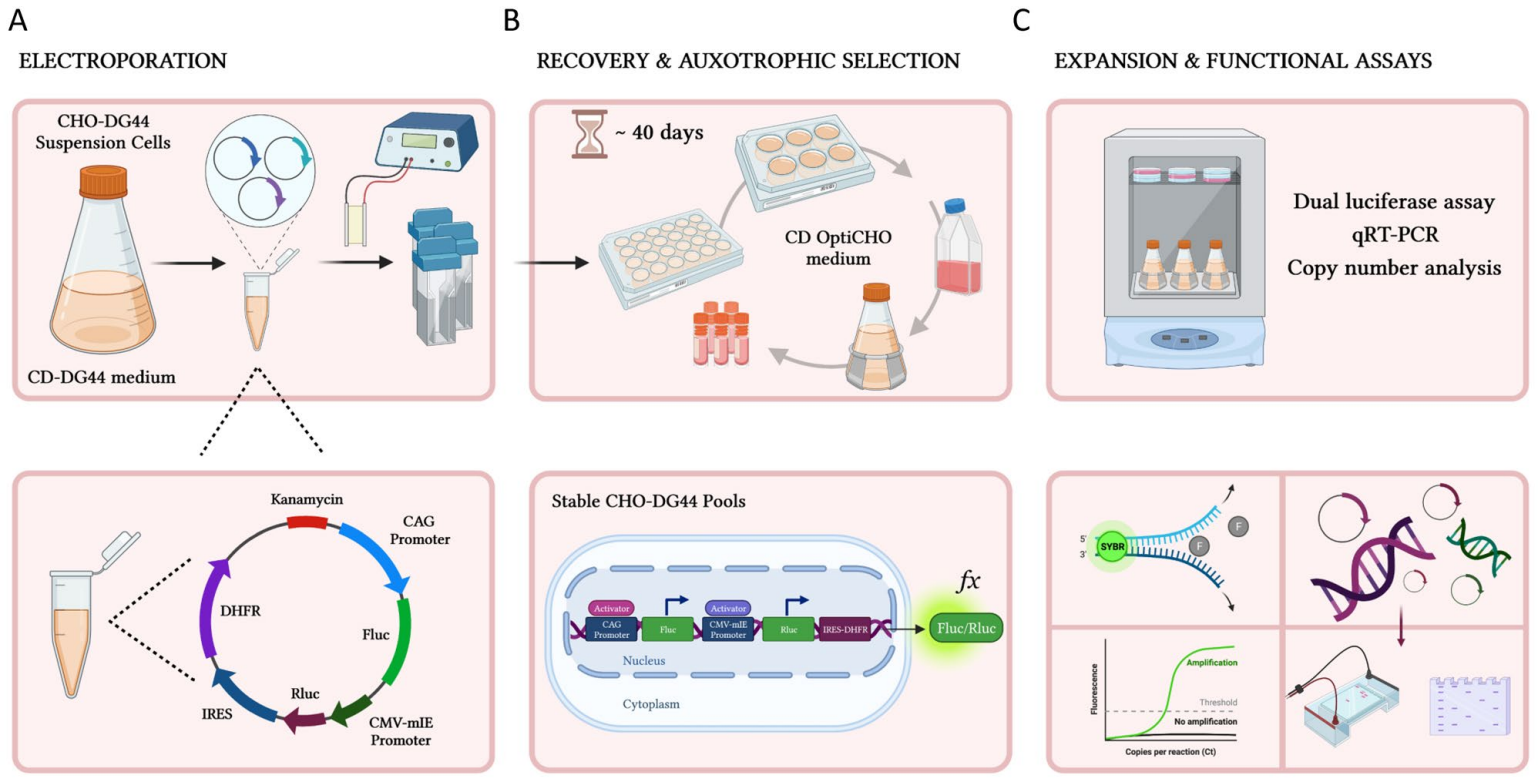
Schematic representation of stable CHO-DG44 cell line development. (**A**) Electroporation: CHO-DG44 suspension culture was electroporated with the vector panel. The constructs harbor variable promoters (the CAG promoter is exemplified in this vector map) upstream of the Fluc coding sequence, followed by the CMV-mIE promoter, Rluc gene, IRES sequence and DHFR gene, as well as the Kanamycin resistance gene as the bacterial selection marker. (**B**) Recovery and auxotrophic selection: Selection was carried out in CD OptiCHO selection media, by sequentially transferring individual CHO-DG44 pools into 6-well plates, T25 flasks and higher volume shake flasks based on their cell division frequency and selection recovery. DHFR based auxotrophic selection took roughly 40 days and cells were occasionally cryopreserved after recovery. (**C**) Expansion and functional assays: After clonal expansion of stable CHO-DG44 pools in HT deprived media, dual luciferase assay, qRT-PCR based gene expression analysis as well as qPCR based copy number experiments were executed. The schematics are created with BioRender.com.

**Figure 5:**
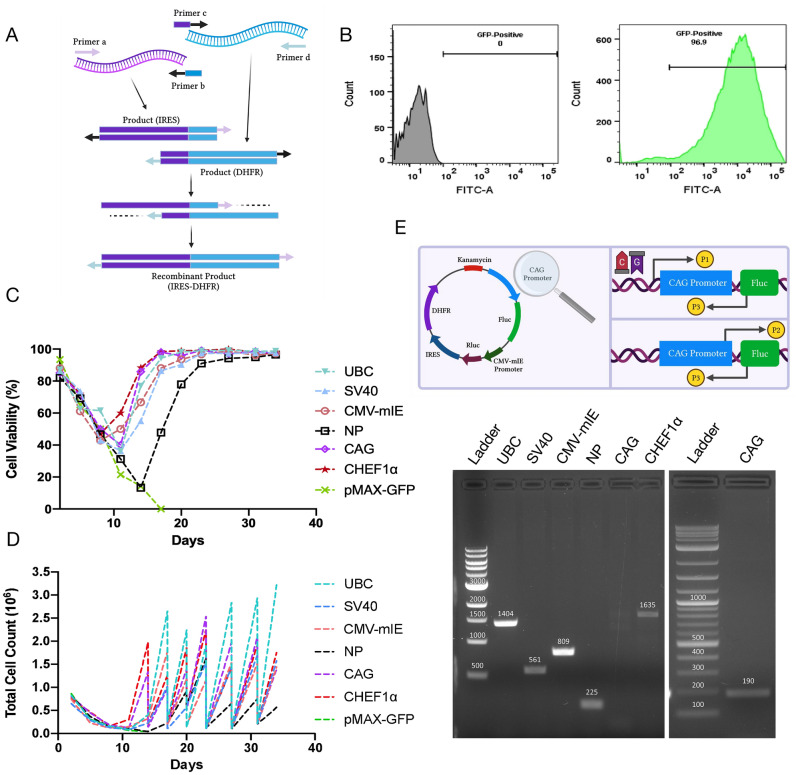
Development and validation of stably transfected polyclonal CHO-DG44 pools. (**A**) Schematic representation of IRES-DHFR cassette cloning using the overlapping PCR method. (**B**) Flow cytometry analysis of electroporated cells after 24 h of nucleofection. pMax-GFP vector was utilized as a transfection efficiency control. GFP positive cell count was detected as 96.9% (on the right). The flow cytometry result on the left indicates untransfected control with 0% GFP positive CHO-DG44 suspension cells. (**C**) Cell viability and (**D**) total cell count percentage during DHFR-based auxotrophic selection. In brief, cells were harvested in HT deprived CD OptiCHO selection media and Trypan blue exclusion-based counting was performed every two to three days. Cells were subcultured at 100.000–200.000 cells/ml. Taking into consideration the growth rate of CHO-DG44 pools, the cells were transferred from multiwell plates to orbital shake flasks. (**E**) The validation of genome integration was performed via conventional non-quantitative PCR using primer sets provided in Table 3. For the CAG promoter, the primer set was altered from P1–P3 pair to P2–P3 that amplifies a shorter fragment with lower GC content (see schematic). Specifically, while P3 reverse primer binds to a region at the 5’ end of the Fluc gene, P2 forward primer targets a region close to the 3’ end of the CAG promoter, that is further verified by Sanger sequencing (data not shown). Note that two different DNA ladders (1 kb DNA ladder—left and 1 kb plus DNA ladder—right) were used in the agarose gel experiments. Expected amplicon and relevant DNA ladder band sizes are highlighted on the agarose gel images. The schematics in (**A**) and (**E**) are created with BioRender.com.

CHO host cell lines can deliver robust and sustained transgene expression, making them the most preferred mammalian cell system for recombinant protein production. In light of this, we explored whether the dual luciferase reporter system validated in transient transfection experiments could be readily applicable and adaptable to analyzing regulatory sequences in stable expression settings. As portrayed schematically in Fig. 4, we conducted a series of experiments in high-density stable transfectants of CHO-DG44 suspension cells, in which the constructs had successfully integrated into their genome. Basically, we selected and prioritized five dual luciferase reporter constructs which displayed relatively strong promoter activity in transient transfection assays (CMV-mIE, SV40, UBC, CAG, and CHEF1α) and modified them accordingly. Using an overlap extension PCR method, we first fused the mouse DHFR gene, an auxotrophic selection marker, downstream of the internal ribosome entry site (IRES) element of the encephalomyocarditis virus (EMCV) (see schematic in Fig. 5A). Taking advantage of the modular design of our vector backbone, we then cloned the IRES-DHFR fragment downstream of the Rluc coding sequence, resulting in a bicistronic expression cassette (Rluc-IRES-DHFR) that permits the coexpression of these two genes from a single transcript, the latter through cap-independent translation (Fig. 1E). This vector panel also included an empty control vector without the variable promoter (NP: no promoter).

The new vector panel was then electroporated into CHO-DG44 suspension cells. Simultaneously, the pMax-GFP vector which encodes the GFP fluorescence protein was used to track nucleofection efficiency. Flow cytometry analysis at 24 h post-transfection revealed that 96.9% of electroporated cells were positive for the GFP signal, indicating a robust transfection efficiency (Fig. 5B). Two days after nucleofection, we applied the auxotrophic selection pressure by culturing the cells in chemically defined commercially available CD OptiCHO media which is naturally devoid of purine precursors (hypoxanthine and thymidine). Every two to three days when sufficient growth was observed, we passaged cells into fresh CD OptiCHO selection media, while transferring them from multiwell cell culture plates to T25 flasks and then orbital shake flasks (see schematic in Fig. 4). During the selection round, we monitored the number of viable and total cell counts at each passage (Fig. 5C,D). As expected, the depletion of untransfected and transiently transfected cells resulted in a rapid and dramatic drop in cell viability and total cell counts. Specifically, we observed that the cell viability decreased from roughly 85–90% to ~ 62% for UBC cells, ~ 43% for SV40 cells, ~ 43% for CMV-mIE cells, ~ 47% for NP cells, ~ 50 for CAG cells, and ~ 47 for CHEF1α cells at day 8 (Fig. 5C). In parallel, total cell counts (per ml) dropped from approximately 0.75–1 × 10^6^ cells to 0.26 × 10^6^ for UBC cells, 0.28 × 10^6^ for SV40 and CMV-mIE cells, 0.3 × 10^6^ for NP cells, 0.36 × 10^6^ for CAG cells, and 0.34 × 10^6^ for CHEF1α cells at day 8 (Fig. 5D). By the end of the second week of selection round, all cell pools except those electroporated with the NP vector developed discernible resistance to selection pressure, as evidenced by an increase in cell viability and total cell count. Meanwhile, the viability of NP cells dropped to a maximum of ~ 13% on day 14. However, on day 17, this value increased to ~ 48%, accompanied by a modest increase in total cell count (Fig. 5C,D). After 3 weeks, cell viability for each CHO-DG44 clone reached about 90–95%, and the cell pools displayed optimal growth characterized by increased viable cell density, indicating that stable transfectant pools had fully recovered from selection pressure (Fig. 5C,D). As predicted, cells electroporated with the pMax-GFP control vector failed to survive the selection pressure and were thus eliminated from the culture (Fig. 5C,D). Next, we sought to confirm that the DHFR-expressing dual luciferase reporter constructs had effectively integrated into the host cell genome. Accordingly, we performed conventional non-quantitative PCR on genomic DNA from all clones using a primer pair that amplifies unique fragments spanning the variable promoter sequences, while sparing the CHO-DG44 genome (see schematic in Fig. 5E). Confirming the genomic integration of each construct, we detected amplicons with expected sizes, particularly, 1404 bp, 561 bp, 809 bp, 225 bp, and 1635 bp for UBC, SV40, CMV-mIE, NP, and CHEF1α, respectively (Fig. 5E). Importantly, no amplification was observed for the CAG fragment, quite possibly due to an inherently high GC content of that promoter (around 65%). To address this issue, we redesigned a new forward primer that was positioned closer to the 3’ end of the CAG promoter. Indeed, the new primer pair supported the amplification of a shorter fragment (190 bp) with lower GC content (roughly 50%), validating the genomic integration of the CAG promoter carrying construct (Fig. 5E).

**Figure 6:**
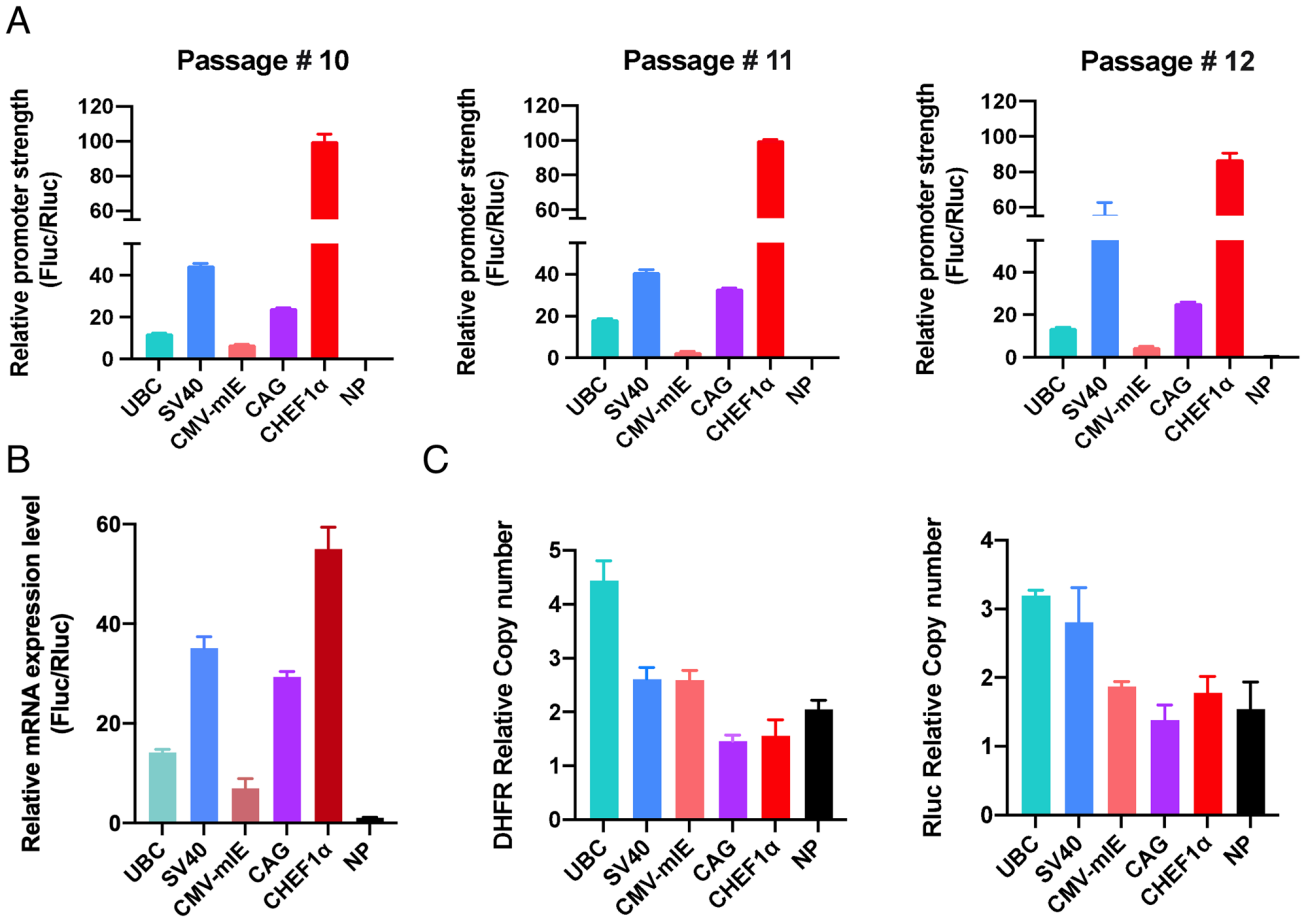
Validation of the vector toolkit in stable transfectants of CHO-DG44 cells. (**A**) Luciferase activity in stable CHO-DG44 pools electroporated with the select panel of vectors (UBC, SV40, CMV-mIE, CAG, CHEF1α and NP). Luciferase assay was carried out in three consecutive passages (Passage 10 to 12), and each promoter activity was concurrently assessed. Quantification of promoter strengths were carried out by dividing the Fluc signal to the Rluc signal. (**B**) mRNA expression of Fluc and Rluc genes was verified by qRT-PCR. Much like the luciferase assay results, CHEF1α yielded the highest transcriptional activity in the stable transfection setting, while CMV-mIE displayed the lowest promoter efficacy. (**C**) Relative DHFR and Rluc copy numbers were calculated according to the internal control gene, GAPDH. Bright Green Master Mix was used for both qRT-PCR assays (**B**) and transgene copy number analysis by qPCR (**C**).

After developing stable transfectants of polyclonal cell pools with resistance to selection pressure, we performed the dual luciferase assay to quantify the activities of distinct promoters. To this end, we collected Fluc and Rluc bioluminescence signals from three consecutive cell culture passages. In doing so, we intended to test and validate the repeatability and reproducibility of quantitative assessment. In a similar way to the transient expression setting, Fluc to Rluc bioluminescence ratio (Fluc/Rluc) was expressed as a quantitative output of promoter strength. Unlike transient transfection experiments, the CMV-mIE promoter displayed the lowest strength (~ 7) in stable CHO-DG44 pools, whereas the CHEF1α promoter had the highest activity (normalized to 100) that was followed by the SV40 (~ 44), CAG (~ 24), and UBC (~ 12) promoters in Passage 10 (Fig. 6A). Intriguingly, we detected slightly varied promoter activities in Passage 11 and Passage 12; SV40 (~ 41 and ~ 54), CAG (~ 33 and ~ 26), UBC (~ 19 and ~ 14), and CMV-mIE (~ 3 and ~ 5), respectively. Nevertheless, the relative order of promoter strength was the same throughout three passages. It is worth noting that the activity of NP construct was undetectable. Collectively, despite spanning a relatively short period of culture time, we detected similar biological outputs in three consecutive passages which infer that reproducible and consistent transgene expression may be obtained with the current collection of promoters. These findings were further corroborated by mRNA expression of Fluc and Rluc genes in Passage 11 samples, which highly correlated with the bioluminescence data, demonstrating that the luciferase enzyme activities were a direct reflection of transcript levels (Fig. 6B). Finally, we analyzed stable transfectants from the earliest passage for integrated transgene copy number, taking GAPDH gene as a reference. These efforts revealed that both Rluc and DHFR genes exhibited comparable yet slightly divergent copy number values ranging from roughly 1 to 4 copies across different clones which likely complement the heterogeneous nature of polyclonal cell pools (Fig. 6C). According to these findings, we conclude that when successfully implemented to coexpress the DHFR gene as a selection marker, the dual luciferase reporter system is reasonably practical and easily adaptable to assessing regulatory sequences in stably transfected CHO-DG44 clones.

## Discussion

CHO cells are currently the primary mammalian expression platform in commercial production of recombinant proteins^2,20^. The demand for biotherapeutics continues to increase, underscoring the importance of steady improvements in prevailing CHO cell line development technologies. Transgene expression cassettes and vectors play important roles in the success of CHO cell systems^8,21^. Thus, the identification and selection of appropriate gene expression regulatory sequences in vector designs is critical. In this study, we developed an all-in-one dual luciferase reporter system in a single backbone that enables systematic and quantitative assessment of regulatory sequences in mammalian cell systems, including CHO and HEK-293T cells. To validate its utility for the intended purpose, we studied a large panel of well-established constitutive promoters. Transient transfection studies revealed that the CMV-mIE promoter exhibited the highest transcriptional activity in CHO cell variants and HEK-293T cells, acknowledging its frequent application in expression vectors designed for strong heterologous protein expression^22,23^. In contrast, HSV-TK, which is generally regarded as a weaker constitutive promoter^24,25^, proved to be the weakest in CHO cells and only marginally stronger than the SV40 promoter in HEK-293T cells.

Intriguingly, the relative order and strength of promoter activities assessed by bioluminescence measurements differed slightly among the cell lines. Despite sharing a common ancestor, CHO cell variants are genetically and phenotypically diverse, with profound differences in fundamental biological and cellular processes^26,27^. For example, the adaptation of CHO cells to suspension growth in serum-free media significantly impacts their lipid metabolism and nucleotide synthesis^28^. Additionally, luciferase reactions are oxidative processes that strongly depend on the energy metabolism of host cells, suggesting that inherent differences in cellular metabolic states are likely to introduce variance into luminescence signals^29^. Aside from intrinsic cellular dynamics, extrinsic factors such as cell culture requirements can also influence metabolic states^30^. Moreover, transcription factor pools, the key determinants of gene expression regulation, may exhibit cell type-specific expression patterns^31–33^. These factors can collectively explain differential activation of reporters and variations in promoter strength estimations. By contrast, the order of promoter strengths calculated using the dual fluorescent reporters was more consistent. This suggests that fluorescent reporters were less affected by the mentioned factors, further consolidating these interpretations. In conclusion, the context-dependent regulation of luciferase reporters by the same promoters may be attributed to inherent differences in the molecular and metabolic features of CHO cell lines, as well as their unique cell culture requirements. Nonetheless, despite their natural limitations, luciferase reporters may still offer several advantages over fluorescent reporters, such as greater sensitivity and lower background signals, justifying the use of both reporter systems based on the specific needs^29,34^.

In a series of additional experiments, we demonstrated the applicability and adaptability of the dual luciferase reporter system in long term culture settings. We identified that the CHEF1α promoter was substantially stronger than other promoters in stable transfectants of CHO-DG44 suspension cells, with CMV-mIE being the weakest of all, a finding that is in complete agreement with prior reports^35,36^. The luciferase calculations were further corroborated at the mRNA level of reporter genes, revealing that the enzymatic readouts were a direct manifestation of promoter transcriptional activity. In addition, we found that the promoter specific activities were consistent and stable during three consecutive cell culture passages, verifying the reproducibility and repeatability of reporter assays in stable clones. Despite only spanning a limited time frame which is an agreeable limitation of stable culture experiments, we maintain confidence that future studies where stable transfectants are cultivated for extended periods will enable investigating the dynamics of transgene expression and the long term intrinsic stability of regulatory sequences. The other possible drawback of the current work is that we intentionally opted to screen promoter activities in heterogeneous pools of polyclonal CHO-DG44 cells rather than single cell derived clones. Although this does not preclude our interpretations, we note that monoclonal CHO-DG44 cells are likely to generate different responses, stressing the significance of further research to reference the relevant processes of recombinant CHO cell line development. Finally, as discussed earlier, only a subset of promoters have been prioritized in stable clone experiments, meaning that additional research will be required to shed light on the activities of remaining promoters that have yet to be explored. Regardless, the existing data sufficiently attests that the dual luciferase reporter system has a great potential to pave the way for the selection of known and the discovery of novel regulatory sequences that may address context-dependent requirements. Developing refined artificial promoters by assembling discrete transcription factor regulatory elements (TFREs) with the choice of promoters that were functionally tested in this study can be a unique application^37^.

We accentuate that the reporter backbones carry SV40 PolyA and BGH PolyA signal sequences already built in downstream of Fluc and Rluc genes, respectively. Select combinations of promoters and polyA sequences may lead to unique transcriptional signatures, emphasizing the need to interpret our findings based on these combinations^38^. That being the case, the reporter system can be modified through further molecular engineering to quantify the strength of other cis-acting regulators of gene expression, including 5’UTR, 3’UTR, introns, S/MARS, and terminators. In line with this, the modular vector backbones may also permit the replacement of current reporter combinations with, for example, secreted bioluminescent reporters, improving the versatility of the reporter system to meet user-defined requirements. Importantly, this unique example may also eliminate cell lysis procedures. Similarly, other positive selection markers conferring resistance to antibiotics, such as puromycin and neomycin, can be incorporated in vector designs, replacing the DHFR gene. Finally, we anticipate that the reporter system might be further adapted to analyze the activities of regulatory sequences at defined genomic loci through site-specific integration of the constructs by next generation genome editing tools such as CRISPR/Cas.

The present study offers several other future implications. In essence, our findings may be useful for future research and development activities including the implementation of select promoters in vector designs for transient or stable transgene expression in CHO cells and perhaps other mammalian (human or non-human) cell lines. In addition, the vector toolkit could be utilized to interrogate the mechanisms limiting cellular productivity including the susceptibility to transcriptional silencing of promoters via multi-site methylation that most studies confront in long term CHO cultures^2,39^. Finally, we also realize that the plasmid backbones might have a potential to serve as base mammalian expression vectors in both transient and stable transfection settings. While enabling the expression of monomeric proteins, the backbones with select combination of promoters may also permit the synthesis of proteins with two domains, such as IgG, and encourage future research to identify optimal heavy and light chain ratios in mAb production.

## Materials and methods

### Cell culture

DHFR sufficient CHO wild type adherent cell line (CHO-WT) was a generous gift from Dr. Gunes Ozhan (Izmir Biomedicine and Genome Center). These cells were grown in DMEM/F-12 medium (11320-033, Gibco) supplemented with 10% FBS (10500064, Gibco) and 1% Penicillin/Streptomycin (15140122, Gibco). DHFR deficient CHO-DG44 adherent cells, obtained as a kind gift from Dr. Lawrence Chasin (Columbia University), were cultured in Alpha MEM Eagle medium (BE12-169F, Lonza) supplemented with 10% FBS and 1% Penicillin/Streptomycin. Both cell lines were maintained at 37 °C and 5% CO_2_ cell culture conditions. CHO-DG44 suspension cells, adapted in-house from CHO-DG44 adherent cells to grow in serum-free suspension culture, were cultivated in CD DG44 medium (12610-010, Gibco) supplemented with L-glutamine at 8 mM final concentration and 18 ml/L (1.8%) Pluronic F-68 Non-ionic Surfactant (24040032, Gibco), and maintained at 37 °C and 8% CO_2_ cell culture conditions and passaged every 3–4 days by dilution. Cells were routinely tested negative for mycoplasma contamination using a PCR-based mycoplasma detection kit (G238, ABM).

### Vector construction

The dual luciferase reporter constructs engineered in this study were developed on the pSF-CMV-Fluc-CMV-BCL2-Sbf1 vector (OG4071, Oxford Genetics) which served as the base plasmid. This original backbone was naturally endowed with dual cytomegalovirus (CMV) major immediate early (mIE) enhancer-containing (CMV-mIE) promoters with the same nucleotide sequence. To engineer the all-in-one dual luciferase reporter system, the BCL2 gene was replaced by the wild type Rluc coding sequence, which was PCR-amplified from the pRL-SV40 vector (Promega) with oligonucleotide pairs listed in Table 1. This new all-in-one vector, named as pCMV-Fluc-CMV-Rluc, coexpressed the Fluc and Rluc reporters. Similarly, we constructed a dual fluorescence reporter system by substituting bioluminescence reporter genes for eGFP and tdTomato fluorescent reporters, respectively. The eGFP and tdTomato coding sequences were PCR-extracted from pEGFP-N1 (Clontech) and pENTR4-tdTomato plasmids (sub-cloned in-house from the pRSET-tdTomato plasmid), respectively (see Table 1 for oligonucleotide sequences). This new reporter system, dubbed pCMV-GFP-CMV-tdTom, provided simultaneous expression of eGFP and tdTomato from a single backbone. Please note that the backbones were labelled with CMV rather than CMV-mIE to simplify nomenclature.

**Table 1:** List of oligonucleotides used in vector construction.

Primer ID	Sequence (5’ → 3’)
SV40-F (**BglII**)	GGCGAC**AGATCT**CTGTGGAATGTGTGTCAGTT
SV40-R (**NotI**)	TAATAT**GCGGCCGC**CGAAAATGGATATACAAGCT
EFS-F (**BglII**)	GGCGCG**AGATCT**TAGGTCTTGAAAGGAGTGGG
EFS-R (**NotI**)	TAATAA**GCGGCCGC**CCTGTGTTCTGGCGGCAAAC
EF1a-F (**BglII**)	TTAAT**AGATCT**CCCGTCAGTGGGCAGAGCGC
EF1a-R (**NotI**)	TGGCA**GCGGCCGC**TATTAGTACCAAGCTAATTC
PGK-F (**BglII**)	TAATAA**AGATCT**GGGTAGGGGAGGCGCTTTTC
PGK-R (**NotI**)	TATTAT**GCGGCCGC**CGAAAGGCCCGGAGATGAGG
TK-F (**BglII**)	GGCGCG**AGATCT**AATGAGTCTTCGGACCTCGC
TK-R (**NotI**)	TAATAA**GCGGCCGC**TTAAGCGGGTCGCTGCAGGG
UBC-F (**BglII**)	TATTAT**AGATCT**GGCCTCCGCGCCGGGTTTTG
UBC-R (**NotI**)	CAGTAT**GCGGCCGC**TCGTCTAACAAAAAAGCCAA
CHEF1a-F (**BglII**)	CATTAT**AGATCT**GGATGGCGGGGCTGACGTCG
CHEF1a-R (**EcoRI**)	CAGGAC**GAATTC**GTTGGATTTGAATTAGCGGT
Rluc-F (**SalI**)	TATTAA**GTCGAC**GCCACCATGACTTCGAAAGTTTATGA
Rluc-R (**SpeI**)	GCAGAC**ACTAGT**TTATTGTTCATTTTTGAGAA
eGFP-F (**NcoI**)	TAATATAGATCTGCCA**CCATGG**TGAGCAAGGGCGAGGA
eGFP-R (**XbaI**)	GGCGCG**TCTAGA**TTACTTGTACAGCTCGTCCA
tdTomato-F (**SalI**)	TATTAA**GTCGAC**GCCACCATGGTGAGCAAGGGCGAGGA
tdTomato-R (**SpeI**)	GGGCGG**ACTAGT**TTACTTGTACAGCTCGTCCA
IRES-F (**XbaI**)	TAGTGT**TCTAGA**TTCCGCCCCCCCCCCCTAAC
IRES-R	GTTCAATGGTCGAACCATGGTGGC/ATCGTGTTTTTCAAAGGA
DHFR-F	TCCTTTGAAAAACACGAT/GCCACCATGGTTCGACCATTGAAC
DHFR-R (**BamHI**)	TCGGCG**GGATCC**TTAGTCTTTCTTCTCGTAGA

After generating these two unique backbones, we designated the upstream CMV-mIE promoter as variable because it was more feasible for molecular engineering of other genetic elements, leaving the downstream CMV-mIE promoter as invariable. Accordingly, the variable CMV-mIE promoter was successfully replaced by the following promoters: simian virus 40 (SV40) enhancer/early promoter (template: pcDNA3.1( +)/myc-His A, Invitrogen), herpes simplex virus thymidine kinase (HSV-TK) promoter (template: pRL-TK, Promega), mouse phosphoglycerate kinase 1 (PGK) promoter (template: TTI-GFP, Scott Lowe laboratory), human eukaryotic translation elongation factor 1 alpha (EF1α) promoter (template: pENTR5’/EF1αp, Invitrogen), human EF1α short (EFS) promoter (template: lentiCRISPR v2, Addgene plasmid #52961), Chinese hamster EF1α (CHEF1α) promoter (template: CHO-WT gDNA, GenBank accession number AY188393.1, position 11151–12623 or − 463 to + 1010 according to transcription start site), cytomegalovirus (CMV) early enhancer element fused to the chicken beta-actin (CAG) promoter (template: pCAG-ERT2CreERT2, Addgene plasmid #13777), and human ubiquitin C (UBC) promoter (template: pLV hUbC-VP64 dCas9 VP64-T2A-GFP, Addgene plasmid #59791). All promoters, except for the CAG promoter, were PCR-amplified from their corresponding templates using oligonucleotide pairs listed in Table 1 and cloned by conventional techniques, i.e. restriction enzyme digestion and ligation. BglII and NotI restriction enzymes were primarily used for cloning of promoter sequences. However, BglII and EcoRI combination was used to clone CHEF1α promoter as it contains NotI restriction site within its sequence (see Table 1 for oligonucleotide sequences). Due to an inherently high GC content, PCR-amplification of the CAG promoter constituted a major challenge. Therefore, a subcloning strategy was adopted. First, it was excised from the pCAG-ERT2CreERT2 plasmid (Addgene plasmid #13777) with SalI and EcoRI restriction enzymes and cloned into the TTI-GFP backbone (from Scott Lowe laboratory), which was precleaved with XhoI and EcoRI enzymes, killing the XhoI site. The resulting vector was restricted with BclI and EcoRI enzymes, releasing a fragment harboring the CAG promoter with compatible sites, which was then cloned into recipient vectors that were predigested with BglII and EcoRI enzymes, destroying the BglII site. The sequence encoding mouse DHFR gene was cloned downstream of the Rluc reporter gene separated by an IRES element, thereby generating a bicistronic transcript encoding Rluc and DHFR as individual polypeptides. Specifically, the IRES sequence was PCR-amplified from the TTI-GFP backbone and mouse DHFR coding sequence was obtained from a mAb expressing template plasmid, kindly provided by Dr. Kasim Diril (Izmir Biomedicine and Genome Center). These amplicons were then assembled by two-step assembly PCR (overlapping PCR), yielding pCMV-Fluc-CMV-Rluc-IRES-DHFR base vector for further manipulation. After finalizing the construction of this vector, the variable promoter CMV-mIE was replaced by SV40, UBC, CAG and CHEF1α promoters.

### Transient transfections

CHO-WT, CHO-DG44 adherent, and CHO-DG44 suspension cells were transfected with the vector panel using Lipofectamine 3000 Transfection Reagent (L3000015, Thermo Fisher Scientific), following the manufacturer’s instructions with slight modifications. Briefly, the transfections were carried out in 48-well cell culture plates using 0.25 μg plasmid DNA mixed with 0.5 μl P3000 enhancer reagent and 0.75 μl Lipofectamine 3000 in 12.5 μl Opti-MEM Reduced Serum Medium (31985062, Thermo Fisher Scientific) per well. The mixtures were incubated at room temperature for 15 min to facilitate stable DNA-lipid complex formation. Finally, 25 μl unique DNA-lipid complexes were directly added to cells in culture medium. Cells were analyzed for luciferase activity 48 h post-transfection.

### Flow cytometry

For the analysis of eGFP and tdTomato expression, CHO-WT, CHO-DG44 adherent, and CHO-DG44 suspension cells were transfected with respective plasmids and maintained in culture for 48 h. Adherent cells were trypsinized, harvested with a complete growth medium and collected by centrifugation. Suspension cells were collected directly via centrifugation. The supernatants were discarded, and the pellets were resuspended in 200 μl DAPI-PBS solution in dark for 2 min. After rinsing, the pellets were resuspended in 100 μl FACS buffer and transferred to FACS tubes. Flow cytometry was run on BD LSR Fortessa and the readings were analyzed by FlowJo V10 software.

### Electroporation

CHO-DG44 suspension culture pools stably expressing transgenes including the mouse DHFR gene were generated by electroporating cells with the Amaxa SG Cell Line 4D-Nucleofector X Kit L (V4XC-3024, Lonza), according to the manufacturer’s instructions. First, the viability and total cell counts of suspension cells which were maintained in CD DG44 medium (12610-010, Gibco) were determined using Trypan blue exclusion test (15250061, Gibco). After collecting cells by centrifugation at 90×*g* for 10 min at room temperature (RT), the pellets were dissolved in Nucleofector solution (1 × 10^6^ cells/100 μl). Cells were then transfected with reporter plasmids selected from transient transfection studies. In brief, cell and plasmid mixture (3 μg, 1 × 10^6^ cells) was transferred to individual nucleocuvettes and were electroporated using the FF-137 program. After electroporation, cells were transferred into 24 well-plates with 1.4 ml of preheated CD DG44 medium for recovery. As a surrogate marker for nucleofection efficiency, CHO-DG44 suspension cells were concurrently electroporated with 2 μg pMax-GFP plasmid, provided by the Nucleofector kit. Flow cytometry was used to assess eGFP expression at 24 h post-nucleofection. To this end, cells were collected into 15 ml falcons and centrifuged at 1200 rpm for 2 min. After discarding the supernatant, cells were labeled with DAPI, resuspended in 50–100 μL PBS and run on BD LSR Fortessa. Flow cytometry data was analyzed using the FlowJo V10 software.

### Auxotrophic selection and CHO-DG44 stable pool generation

Auxotrophic selection was initiated two days after electroporation. Specifically, recovered cells were harvested by centrifugation and resuspended in CD OptiCHO medium (12681011, Gibco) supplemented with 1.8% Pluronic F-68 and 8 mM L-glutamine and transferred into 6-well culture plates. Selection was sustained in CD OptiCHO selection media by successively transferring the cells into T25 flasks and high volume shake flasks according to their division frequency and growth rate. During the selection process, cells were kept in 6-well plates for as long as 17 days (except for the NP vector electroporated cells). Unlike other clones, the recovery of NP vector electroporated cells took 27 days. Once the cells recovered selection, emerging clones with considerable growth were successively transferred into 20 ml CD OptiCHO selection media in 125 mL polycarbonate rlenmeyer shake flask with vent cap (431143, Corning) and cultured at 37 °C and 8% CO_2_ cell culture conditions. Celltron orbital shaker platform (Infors HT) at an agitation rate of 130 rpm was used for shaking. Every 2–3 days, cells were collected via centrifugation (1200 rpm, 5 min) and subcultured at a 100.000–200.000 cells/ml density following Trypan blue exclusion counting. To track the selection process, pMax-GFP electroporated cells were employed as a reference control.

### Dual luciferase assay

The Dual Glo Luciferase luminescent assay (E2920, Promega) was carried out in accordance with the manufacturer’s protocol with slight modifications. Detailed protocols for transient transfection and stable culture settings are described here. Following transient transfections in CHO-WT and CHO-DG44 adherent cells, cell culture media was discarded, and cells were rinsed twice with PBS. For CHO-DG44 suspension cultures, cells were collected into 1.5 ml eppendorf tubes by centrifugation and rinsed twice with PBS, repeating centrifugation at each step. The Dual Glo Luciferase Reagent (25 μl/well) was diluted in 25 μl/well PBS. The mixture was added to each well or eppendorf tube for suspension cells, inducing cell lysing and the Fluc activity. After incubating for 10 min at room temperature, lysates (50 μl) were transferred into 96-well flat bottom white polystyrene plates (3917, Corning). Fluc activity was measured using Centro XS3 LB 960 Microplate Luminometer (Berthold Technologies). Then, the same volume of Dual Glo Stop&Glo reagent (diluted in Dual Glo Stop&Glo buffer at a 1:100 ratio) was added to each well and incubated at room temperature for 10 min, quenching the Fluc signal and inducing luminescence for Rluc which was measured using the same luminometer. For CHO-DG44 suspension cultures stably expressing the reporter genes, cells were collected into 15 ml falcon tubes and centrifuged at 1200 rpm for 5 min. After rinsing twice with PBS, the same protocol was applied. In both settings, promoter strength was calculated by dividing the Fluc luminescence to the signal emitted by the Rluc after subtracting the relevant background measurements of each luminescence recorded from non-transfected cells.

### RNA extraction and quantitative reverse transcriptase PCR (qRT-PCR)

Total RNA extraction was performed using Macherey–Nagel NucleoSpin RNA kit (740955, MN), according to the manufacturer’s protocol. RNA quantities and purities were assessed by NanoDrop 2000 (Thermo Scientific). First strand cDNA synthesis was carried out with iScript cDNA Synthesis Kit (1708890, BioRad), according to the manufacturer’s protocol with slight modifications. Specifically, 2 μg RNA was converted to cDNA in a 40 μl reaction using 5X iScript reaction mix supplemented with oligo(dT) and random hexamer primers and 2 μl iScript Reverse Transcriptase. qRT-PCR reactions were set up at ABI Prism 7500 Fast Real Time qPCR machine using Bright Green 2X qPCR Master Mix (MasterMix-S-XL, ABM). For each reaction, 0.8 μL primer-mix (see Table 2 for oligonucleotide pairs), 5 μl master mix and 4.2 μl cDNA (25 ng) were used. qRT-PCR data were analyzed using the 2^-ΔΔCt^ method. CHO specific GAPDH was used as the housekeeping internal control.

**Table 2:** List of oligonucleotides used in qRT-PCR assays.

Primer ID	Sequence (5’ → 3’)
Fluc-F	GCCATGAAGCGCTACGCCCTGG
Fluc-R	TCTTGCTCACGAATACGACGGTGG
DHFR-F	ACCAGGCCACCTCAGACT
DHFR-R	GAGAGGACGCCTGGGTATT
GAPDH-F	CACTCTTCCACCTTTGATGCTG
GAPDH-R	GTCCACCACTCTGTTGCTGTAGC
Rluc-F	GGAATTATAATGCTTATCTACGTGC
Rluc-R	CTTGCGAAAAATGAAGACCTTTTAC

### Genomic DNA extraction and non-quantitative polymerase chain reaction (PCR)

Genomic DNA isolation was performed by EZ-10 Spin Column Blood Genomic DNA Miniprep Kit (SK8253, BioBasic), according to the manufacturer’s instructions. DNA quantities and purities were assessed by NanoDrop 2000. Conventional non-quantitative PCR was performed using Q5 High-Fidelity DNA Polymerase (M0491, NEB) and designed primer sets (see Table 3) according to the manufacturer’s protocol. Unlike those with normal GC content levels, PCR reactions for CAG and CHEF1α promoters with high GC content were supplied with Q5 High GC Enhancer.

**Table 3:** List of oligonucleotides used in conventional RT-PCR assay.

Primer ID	Sequence (5’ → 3’)
Promoter-F (P1)	TCAATCGTTGCGTTACACAC
Promoter-R (P3)	AGAATGGCGCTGGGCCTTTC
CAG (P2)	GGTTCGGCTTCTGGCGTGTGACC

### DHFR copy number analysis

Genomic DNA of relevant clones was examined by quantitative PCR (qPCR) to assess the copy number of DHFR gene integrated into the CHO-DG44 genome. Briefly, reactions were set up at ABI Prism 7500 Fast Real Time qPCR machine using Bright Green 2X qPCR Master Mix. In each reaction, 0.8 μL primer-mix, 5 μL master mix and 4.2 μL gDNA (in total ~ 100 ng) were used. QPCR data were analyzed using the 2^-ΔΔCt^ method. Near-diploid CHO-DG44 genome is estimated to harbour 176 copies of the GAPDH gene^40^. The copy number of the DHFR gene for each CHO-DG44 stable clone was normalized relative to the GAPDH gene and the 176 number was used as the reference standard.
